# The Well-Being of the Elderly: Memory and Aging

**DOI:** 10.3389/fpsyg.2020.00778

**Published:** 2020-05-26

**Authors:** Juan José Maldonado Briegas, Ana Isabel Sánchez Iglesias, Sergio González Ballester, Florencio Vicente Castro

**Affiliations:** ^1^Financial Economy and Accounting Department, University of Extremadura, Badajoz, Spain; ^2^Evolutionary and Educational Psychology Department, University of Burgos, Burgos, Spain; ^3^Association of Developmental and Educational Psychology for Children, Youth, Elderly and Disabled People (INFAD), Badajoz, Spain

**Keywords:** well-being, cognitive development, longevity, memory, health

## Abstract

The world population increases every day as a consequence of the increase in life expectancy and longevity of humans. There are several factors analyzed in the different studies that have been developed on this topic. The research carried out in this field distinguishes biological, cultural, and cognitive factors; some of them describing similar results, while many others showed antagonistic results. Our study was oriented to the accomplishment of a bibliographical revision with the objective to verify the scientific production on “memory, cognitive development, and aging linked with longevity”—international/ national studies were analyzed and identified. The method carried out was through a research in the databases: SciELO, UAM, PePSIC, LILACS, PubMed, PsycINFO, Dialnet, and Teseo; in a period of 10 years, considering the studies published between January 2008 and December 2017. From the results found at first, 16 articles were analyzed after the application of the exclusion criteria. Likewise, we analyzed the relationship of longevity with the level of studies in Spain from a group of people over 60 years of age counted in January 2017. The literature review determined that there are psycho-cultural aspects that have a decisive influence on the increase in longevity, such as the performance of activities with positive mental states, positive emotions and experiences, and the level of studies.

## Introduction

The historical evolution of the world population has been one of gradual and constant growth, with fluctuations in these growths, increasing significantly since the middle of the last century as a result of advances in technology and, therefore, in the field of medicine. In fact, people can live on average up to an age range between 80 and 120 years today. This might be due to improvements in living conditions (physical activity, diet, no smoking, etc.), to care medical, as well as cognitive development which are aspect that we consider as one of the most significant and influential variables in the increase of the longevity of the population.

Spain is one of the longest living countries in the world. With more than 100,000 people of 100 years or more, Spain is the country with the highest life expectancy, after Japan, according to OECD data and the latest data from the population census, and data from various analyzes of the year 2017. Average life expectancy at birth is 83.2, somewhat below the average of 83.4 years of average life that Japanese can expect to live.

This aging of the population is one of the results of the evolution of demographic change components of the decrease in mortality, as well as of the increase in life expectancy and the decrease in birth rate. Therefore, population growth and age composition is influenced by these changes. In fact, to the extent that demographic variation advances and there are decreases in mortality and fertility fundamentally, there is a gradual process of population aging (Chackiel, 2006).

If Spain continues the current health trends and maintains its health potential, in 2040, it will go from being the fourth longest life expectancy country to being the first.

As of January 1, 2018 in Spain, there are 8,908,151 elderly people, 19.1% of the total population (46,722,980); and they continue to increase, both in number and in proportion. The average age of the population is 43.1 years; about 10 years above if we compare it with the 70s, where the average age were 32.7.

On the other hand, the proportion of octogenarians continues to grow to a greater extent; representing 6.1% of the entire population, and will continue to gain weight among the elderly population in an aging process of the elderly. In fact, the number of centenarians begins to be relevant; there are currently 11,229 registered voters.

This fact has led us to study the longevity of people and their relationship with the level of studies carried out throughout their lives, observing interesting results:

According to data from the INE^1^ regarding the “population aged 16 and over by level of education attained, sex, and age group” in mean values for the year 2018, divisions have been established at an interval, from 16 to over 70 years.

The different types of educations that have been analyzed for each of the classes (previous intervals, from 16–19 to >70 years) for the mean values of the year 2018 have been stated below:

- Illiterate
- Incomplete primary studies
- Primary education
- First stage of Secondary Education and similar
- Second stage of secondary education, with general orientation
- Second stage of secondary education with professional orientation (includes postsecondary education not superior)
- Higher education.

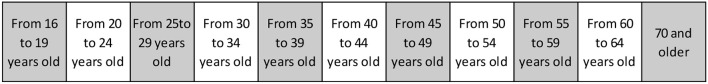


**Figure 1:**
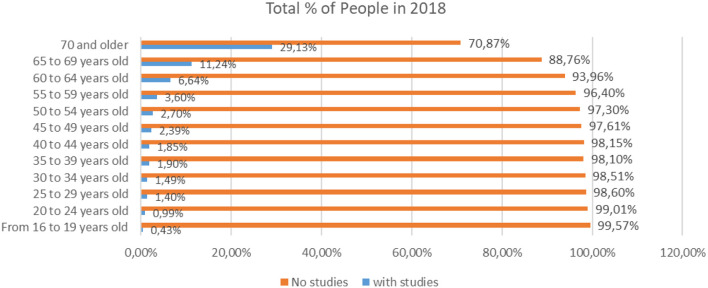
Percentage of people with and without studies in different age scales for the year 2018 (source: own elaboration based on INE data).

The Comparative Analysis (Figure 1) between people who do not have studies (illiterates and people with incomplete primary education), and people who, if they have studies for the mean values of 2018 according to data from the INE, reflects that the percentage of people who have studies is over 70% in people over 70 years, 88.76% in those between 65 and 69 years, and above 93% in people under 65 years.

**Figure 2:**
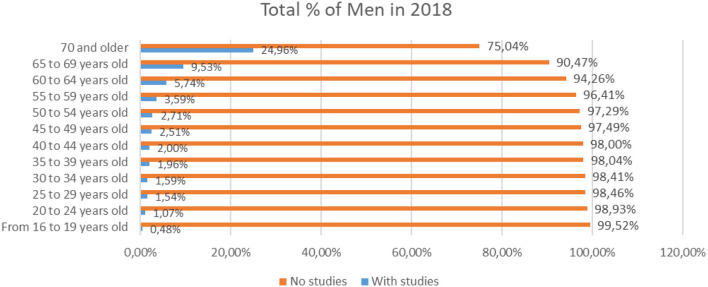
Percentage of men with and without studies in different age scales for the year 2018 (source: own elaboration based on INE data).

Also, in the same analysis of studies based on sex (Figure 2), we observe that 75.04% of men of 70 or more years have studies, compared to 24.96%, who did not finish their studies.

**Figure 3:**
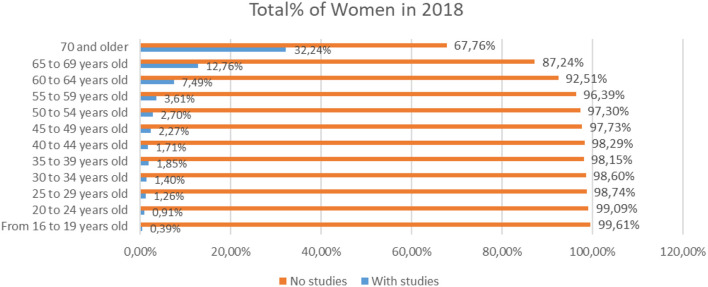
Percentage of women with and without studies in different age scales for the year 2018 (source: own elaboration based on INE data).

On the other hand, women in the age of 70 years or older, 67.76%, have studies compared to 32.24% which did not finish their studies (Figure 3).

**Figure 4:**
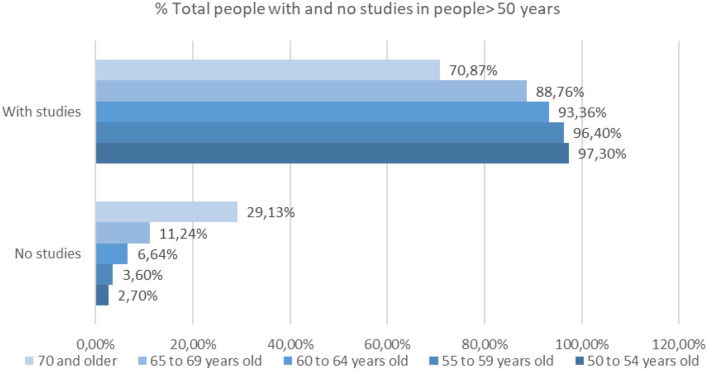
Percentage of people with and without studies in people over 50 years of age (source: own elaboration based on INE data).

The data collected by the INE have allowed us to perform the analysis of the percentage of people who have made a study throughout their lives compared to those who have not done any. The following (Figure 4) shows a comparison between the number of people in % who have done any study of any level as well as the number of people who have not done any study or have not completed it, in ages over 50 years.

**Figure 5:**
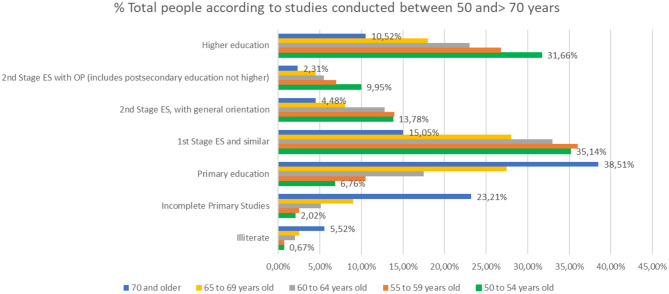
Percentage of people over 50 with different levels of education (source: own elaboration based on INE data).

The distinction between the different levels of studies (Figure 5) for those who finished their studies is significant, since those people over 70 with higher education levels surpass those in the Second Stage of secondary education with job or general orientation.

**Figure 6:**
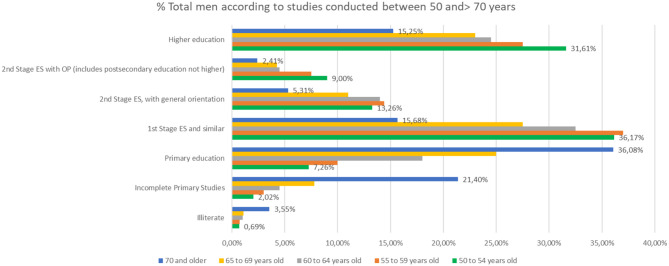
Percentage of men over 50 with different levels of education (source: own elaboration based on INE data).

**Figure 7:**
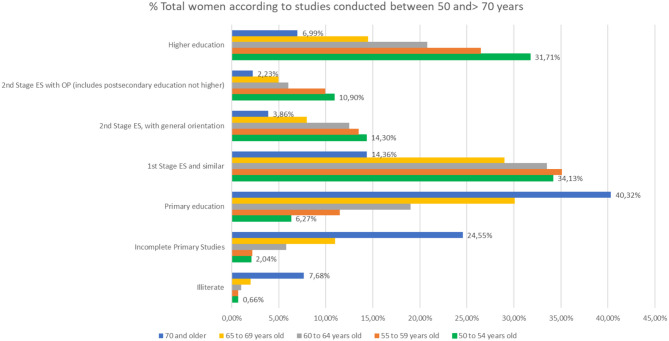
Percentage of women over 50 with different levels of education (source: own elaboration based on INE data).

The difference in the level of studies in men and women becomes relevant in people older than 65 years of age (Figures 6, 7). In people between 65 and 69 years, the percentage of people with higher education is 22.4% in men and 13.3% in women, while in people over 70, 15.5% of men has a higher education compared to 6.6% of women.

The current data indicate that older people have tripled in relation to the total population. Notwithstanding, these data must be interpreted in light of other sociodemographic and sociopsychological variables.

The different demographic studies show an immovable reality because there is a greater longevity of the population in relation to the totality of the population. However, other variables must be considered together with it, such as: the decrease of the birth rate; the almost eradication of infant mortality; the improvement in hygienic conditions; the improvement in the quality of life; the advances in medicine in general; the advances in gerontology in particular. This has made our study a progressive development in the levels of studies and the corresponding cognitive development.

In this sense, some studies (Steverink et al., 2001) describe the influence of sociodemographic variables, indicating that people with better subjective health, higher income, less feeling of loneliness, higher educational level, and greater optimism would tend to experience a healthier aging process in terms of continuous development than of physical deterioration or social losses. This healthy aging in older people is linked to a higher emotional well-being that would favor a better aging experience (Prieto-Flores et al., 2008).

On the other hand, the social psychological variables would be those related to Ryff's multidimensional model: self-acceptance; positive relationships with other people; autonomy; domain of the environment; purpose in life; and personal growth. Ryff describes psychological well-being as “the effort to perfect one's own potential, thus it would have to do with life acquiring meaning for oneself, with certain efforts to overcome and achieve valuable goals; The central task of the people in your life is to recognize and make the most of all your talents. He stressed the responsibility of the individual to find the meaning of his existence, even in the face of harsh or adverse realities.”

It must therefore be understood that psychological well-being as personal construction rests on the psychological development of each individual who has the ability to interact harmoniously with their environment (Ortiz Arriagada and Salas, 2009).

For older adults, self-efficacy carries a meaning far beyond the overcoming of tasks of the physical order. Its importance is associated with the feeling of being able to participate in the solution of conflicts that arise in daily life, providing solutions, and all this allows them to create coping strategies necessary to deal with adversities (Ortiz Arriagada and Salas, 2009).

The fact of aging is a process inherent to the passage of time that involves certain transformations and vital transactions in the individual. The course of this process, as well as its consequences, is modulated on the one hand by personal variables and on the other by those derived from a broader psycho-socio-cultural context fundamentally (Borrell et al., 2011).

In this sense, Gerontology and the Psychology of Aging aims to describe and explain aging and intervene in the specific problems of the elderly in order to increase their well-being and improve their quality of life. To that objective, we can add that preventing and intervening in those factors are being demonstrated as propitiators of greater longevity and a higher quality of life.

Cognitive functions are all mental activities that the individual performs when interacting with the surrounding environment. From this perspective, they represent the essence of personal adaptation of the individual and of all social progress due to the ability of human beings to develop strategies and plan the future to evaluate their consequences (Bromley, 1988; cited in Muñoz, 2002).

In old age, there are deteriorations in the processing, learning, and recovery of new information, problem solving, and speed of response. Thus, it is known that one of the most frequent problems in aging is the decline of memory. In fact, the subjective complaint of lack of memory appears in 70% of elderly subjects (Laurent et al., 1997), but performance improves markedly when they are given clues that guide their memory (Davis and Mumford, 1984). In this sense, various studies (Molina et al., 2012) conclude that the realization of intellectual activities and the maintenance of cognitive functioning are two entities that are associated in the very elderly, in the absence of cognitive impairment, that is; the decrease in cognitive performance that occurs associated with age will be less pronounced for those more mentally active people.

Furthermore, the signs of aging can be identified in one's own body or in one's own mind. The signs of aging at the body level are manifested in the following realities: decrease in body muscle mass and increase in fat; there is also an increase in pigmentation in some tissues and in the interconnections of some molecules such as collagen; changes in glomerular filtration rate, maximum heart rate, vital capacity, and other measures of functional capacity; reduction of the capacity to respond adaptively to variations that occur in the environment; increased vulnerability to diseases; increase in mortality with age; etc. (Maldonado-Briegas et al., 2019).

On a psychological level, the signs of aging are identified by another set of variables closely related to cognitive development, personality, social activities, etc. and in the negative aspect mainly with memory loss. In this sense, the art of aging offers us a series of guarantees to live longer and live happier lives (Martin and Espanola, 2019).

All these aspects, both internal and external to the person, are what have led us to carry out an in-depth analysis of the factors that have a determining influence on the increase in longevity, variables related to the conservation of cognitive capacity, memory, and the aging process, and the level of studies.

For this, we started from the bibliographical study carried out by Lucchese et al. (2018)—“The reality of memory in healthy elderly and aging MEMORY, AGING, AND LONGEVITY,” which has allowed us to know the different studies carried out on memory, aging, and longevity, and analyze its link with the level of studies.

## Recent Studies on Longevity: Theoretical Framework

With the aim of offering an effective scientific contribution to the positioning of “*cognitive development and longevity,”* it was necessary to proceed to a verification of what has been done in terms of research *on “memory, aging, cognitive development and longevity;”* that is, to consider the state of the art, not only to capture the scientific production already made, but also to clarify the problem and to have the collaboration of researchers who point out possible gaps in their studies, doubts, and controversies that persist.

To do this, we conducted a literature review in different databases, such as: SciELO, UAM, PePSIC, LILACS, PubMed, PsycINFO, Dialnet, and Theseus, in a period of 10 years, from January 2008 to December 2017.

First, the descriptors “memory” and “aging” were found in 54 articles in the SciELO, in UAM 746, in PePSIC 19, in LILACS 149, in Dialnet 417, and in Teseo 1. For the same descriptors in English, “Memory” and “aging” used in PubMed and PsycINFO databases were 21,090 and 1964, respectively.

Secondly, the research was carried out with the descriptors “cognitive development” and found in the SciELO 586 articles, in the UAM 6414, in the PePSIC 10, in the LILACS 882, in the Dialnet 7715, and in the Teseo 16; and the Anglo-Saxon terms “cognitive” and “development” were found in PubMed database 58055, and in PsycINFO 10337.

As the number of articles in both descriptors was very large, we opted for a new revision and two new searches were carried out by adding to the descriptors. The descriptor “longevity” was used so that the descriptors used in the analyzed search were “cognitive development and longevity” and “memory, aging, and longevity” for the SciELO, UAM, PePSIC, LILACS, Dialnet, and Theseus databases; and “cognitive development and longevity” and “memory, aging, and longevity” for the PubMed and PsycINFO databases. With these additions, the following results were found:

For the descriptor “*cognitive development and longevity”* and “*cognitive development and longevity,”* the database SciELO, PePSIC, LILACS, and Theseus were zero(0), in the UAM, there were 74, in Dialnet 17, in PubMed 201, and in PsycINFO 11.

For the descriptor “*memory, aging, and longevity”* and “*memory, aging, and longevity”* the database SciELO, PePSIC, and Teseo were zero(0), in the UAM, 48 articles, for LILACS 1, for Dialnet 9, for PubMed 420, and for PsycINFO 12.

Regarding these results, which would make work in UAM viable, but which, in turn, would eliminate two databases (PePSIC and SciELO) and greatly restrict the number of articles in two others (PsycINFO and PubMed), there was an option that was made to consider the search with two and three descriptors in Spanish in the databases (UAM, Dialnet, LILACS) and with two and three descriptors in English in the databases (PsycINFO, PubMed), with the aim of having a broader vision of the production of the subject studied. The articles went through an analysis process which first excludes duplicates and subtracts 53 for the analysis. The second aspect is based on the reading of the title and summary. However, this was not enough to decide the inclusion or exclusion because the title did not represent the core of the article or the summary was very succinct and left doubts as to the relevance of the inclusion, samples, results, and conclusion that were also analyzed. For the exclusion: the criteria were also used: articles not available in full free of charge, duplicates, and revision.

For inclusion: the criteria were used: study regarding the proposed topic (memory research or cognitive development, aging and longevity with or without intervention proposal), sample constituted by healthy elderly, and period of 10 years. The selected articles were read in their entirety to elaborate a comparative analysis and detect its main aspects, similarities and differences, peaceful points and controversies about the association of memory (and/or cognitive development), aging, and longevity.

Table 1 contains the flow diagram elaborated, where the process of searching and selecting the material for analysis is condensed, showing it in a synthetic way and allowing a global vision of it.

**Table 1:** Percentage (%) of the age of death of human populations throughout different periods.

		**Young people**	**Adults**	**Old**	**Seniles**	
		**0–20**	**21–40**	**41–60**	**61–x**	
Australopithecus	(n. 173)	58.4	41.6	–		Mano 1975v
Homo erectus	(n. 33)	48.5	21.2	30.3	–	Weidenreich 1943 et 1951
H.s neanderthalensis	(n. 39)	48.7	41.0	10.3	–	Vallois 1960
H.s sapiens						Fusté 1954
Paleolithic (sup.)	(n. 76)	54.0	34.2	11.8	–	Vallois 1960
Mesolithic	(n. 71)	38.2	57.8	3.5	0.7	Vallois 1960
Néo-Entolithic	(n. 101)	39.6	41.6	17.8	1.0	
Bronze (Autriche Mer)	(n. 273)	24.2	39.9	28.6	7.3	Vallois 1960
Western mer. (1829 AD)		54.0	12.2	12.8	21.0	Vallois 1960
Western (1927 AD)		18.1	11.9	22.6	47.4	Vallois 1960

*Life curve of Homo erectus and current populations (taken from Alciati, 1987) (2)*.

The analysis of the results offered different questions discussed, clearly differentiating qualitatively two genetic and non-genetic variables linked to longevity:

### Longevity and Genetic Influence

We studied that the variables cited in the articles influencing longevity could be of a genetic base which results in few works focused on non-psychological aspects, although sometimes interconnected with psychological ones. The results were the following:

The longevity of the parents is associated with a better aging of the brain in the middle-aged children according to Murabito et al. (2013) and Dutta et al. (2014) who investigated the aging of the children of long-term parents.

The offspring of non-agenarians (genetic) with a family history of longevity have a better cognitive performance compared to the group of their partners of comparable age according to the study by Stijntjes et al. (2013).

According to Barral et al. (2013), they stated that genetic variants influence memory performance in long-lived families after analyzing the performance of memory in the Long Life Family Study. This aspect is also a non-genetic variable.

The Bio-Socio-Sanitary variables (Doing sports, healthy eating, not smoking, etc.) contribute to longevity (Franco et al., 2018) as well as to Cognitive development (Conf. Cephalal et Neurol, 2018, Vol. 28, N. 1: 5–15).

### Longevity Results Due to Non-genetic or Socio-Psychological Influence

There are other factors that influence the longevity of people; they are non-genetic factors or also called socio-psychological. These factors influence in one way or another the increase in longevity, as determined by the results of various studies conducted in this field.

Thus, if we focus on the effects of a lifestyle committed to cognitive vitality (Stine-Morrow et al., 2008), it is concluded that the commitment to training specific skills can mitigate cognitive declines related to age.

Furthermore, Barral et al. (2012) conclude in their study that cognitive performance can serve as an important endophenotype for longevity by studying cognitive function in families with exceptional survival. Precisely, a greater sense of purpose is related to a greater probability of survival and longevity (Windsor et al., 2015).

Along the same lines of reasoning, a daily cognitive occupation would protect against cognitive impairment. This was stated by Zhu et al. (2017) after they conducted a longitudinal study in Chinese older adults on leisure, education, and cognitive impairment activities in Adults. According to this study, recreational activities protect against cognitive deterioration among Chinese elders, and the protective effects are deeper for educated elders.

Likewise, participating in learning new skills improves episodic memory in older adults, improves cognition in relation to participation in social or challenging activities, and helps to live longer (Chan et al., 2016). Although the ability to solve daily problems differs with age, along with the underlying processes, this increases longevity (Chen et al., 2017).

Other authors (Olchik et al., 2012) state that memory training is a feasible non-pharmacological intervention that could bring a positive change in performance in older adults facing cognitive impairment. They demonstrated how memory training (MT) in mild cognitive impairment (MCI) generated changes in cognitive performance; thus, Lipton et al. (2010) affirm that factors associated with longevity can protect against dementia and Alzheimer's disease. On the other hand, Miller et al. (2013) studied the effect of computerized brain exercises on the elderly; Learning new things and keeping the mind engaged can be an important key to successful cognitive aging, as suggested by popular wisdom and our own intuitions (Park et al., 2014). A lower level of logical abstract thinking and a slower information processing speed are associated with shorter survival among adults (Nishita et al., 2017).

On the other hand, it should be borne in mind that positive behavior in daily life activities represents several dimensions of personal well-being, health and safety, and may confer greater longevity (Chang et al., 2012). Thus, older people who buy every day to meet their needs have a 27% lower risk of death than less frequent shoppers. Similarly, extensive social participation, as well as regular participation in group leisure activities, or in organized social activities and informal social interactions in particular can have beneficial effects on the functional health of older adults through behavioral and psychosocial pathways (Gao et al., 2018).

Finally, a relevant result in the field of longevity is that contributed by Ritchie et al. (2013) who affirm that the increase in education significantly improves the cognitive abilities of later life.

Extensive social participation, regular participation in group leisure activities, organized social activities, and informal social interactions in particular can have beneficial effects on the functional health of older adults through behavioral and psychosocial pathways, Gao et al. (2018).

### Results of the Analysis of the Articles Related to Longevity From a Socio-Psychological Influence Point of View

Field experimental studies on cognitive training were conducted in Stine-Morrow et al. (2014) and Murabito et al. (2013) examined the relationship between longevity, parental cognition, and subclinical brain aging markers in a sample of 728 individuals; Dutta et al. (2014) tested the association between the longevity of parents and the cognitive impairment of old age through a biennial evaluation for ages between 64 and 79 years. Windsor et al. (2015), in a sample of 1475 older adults, examined associations of individual differences in purpose sense with levels and rates of change in aging rates (health, cognition, and depressive symptoms). Chen et al. (2017) examined age differences in the relative contributions of fluid and crystallized skills to solve everyday problems in a sample of 221 healthy adults between the ages of 24 and 93. Chan, Haber, Drew, and Park studied the influence and benefit of cognition and the improvement of daily function by training with the use of tablet, computer, and associated software applications in a total sample of 54 older adults (60–90 years old). Olchik et al. (2012) conducted a randomized controlled trial where memory was studied through cognitive tests in three different intervention groups. Stijntjes et al. (2013) studied a sample of 500 individuals whether children of non-generic siblings with a family history of longevity perform better on cognitive tests compared to their peers as controls. Festini et al. (2016) examined the relationship between activity and cognition in adults between 50 and 89 years old, in a sample of 330 people, by completing a cognitive battery and a questionnaire of environmental demands of Martin and Park (MPED) and performing an evaluation of the activity. Miller et al. (2013) conducted research to explore whether computerized brain training exercises improve cognitive performance in older adults, using a sample of convenience by randomly assigning an intervention group (*N* = 36), which used a computer program 5 days a week for 20–25 min each day, together with a waiting list control group (*N* = 33). Park et al. (2014) studied whether sustained participation in learning new skills that activated working memory, episodic memory, and reasoning over a period of 3 months would improve cognitive function in older adults. Nishita et al. (2017) also studied in a sample of 1,060 individuals the longitudinal relationship between cognitive abilities and subsequent death in Japanese elderly people. Zhu et al. (2017) examined the association between leisure time activities and the risk of developing cognitive impairment among Chinese elderly people, and investigated whether the association varies according to educational level, using a sample of 6,586 participants. Furthermore, Ritchie et al. (2013) analyzed in two longitudinal cohorts the association between education and cognitive change for life. Chang et al. (2012) analyzed in a sample of 1,841 representative free-standing Taiwanese people, purchasing behavior. Gao et al. (2018) studied the effects of participating in different types of social activities at the onset of functional disability and the underlying behavioral and psychosocial mechanisms among adults aged 65 and older in China through a health longevity study during the years 2005, 2008, and 2011.

### Methods

Different methods were used to test the association between the longevity of the parents and the measurements of cognition and brain volumes. Murabito et al. (2013) used multivariable linear and logistic regression to adjust age, sex, education, and time for NP or brain MRI testing, resulting that the association with hyperintensity of the white matter was no longer significant in models adjusted for cardiovascular risk factors and disease.

Dutta et al. (2014) conducted the study classifying the descendants into groups of parental longevity based on gender-specific distributive cut-off points, using for that purpose covariable models that included race, education of the respondents, and income status during childhood and Adulthood. Düzel et al. (2016) used models of structural equations in the validation of a new self-report measure; the Subjective Health Horizon Questionnaire (SHH-Q). The SHH-Q evaluated the perspectives of future time of individuals in relation to four interrelated but distinct lifestyle dimensions: (1) novelty-oriented exploration (Novelty), (2) physical fitness (Body), (3) Work objectives (Work), and (4) Life goals (Life goals). Four studies developed cognitive batteries: Chen et al. (2017) performed a cognitive battery to measure fluid capacity (processing speed, working memory, inductive reasoning) and crystallized capacity (multiple measures of vocabulary), and predicted performance in the test of everyday problems (EFA) by arriving at the result that the main predictor of performance in solving everyday problems for young adults was fluid capacity; noting further that crystallized capacity became the dominant predictor with increasing age; On the other hand, Chan, Haber, Drew, and Park, performed a cognitive battery in a sample of 54 older students by analyzing the memory of episodes and processing speed, mind control, and visuospatial processing. Festini et al. (2016) carried out a cognitive battery and a questionnaire of environmental demands of Martin and Park (MPED), on a sample of 330 adults of 50 and 89 years, to study the relationship between activity and cognition. The results showed that greater activity was associated with better processing speed, working memory, episode memory, reasoning, and crystallized knowledge. In turn, they demonstrated that living a busy lifestyle is associated with better cognition. Park et al. (2014) performed a cognitive battery and psychosocial questionnaires before and after the intervention on a sample of 211 individuals between the ages of 60 and 90 years. The evaluators were blinded to the assignment of the condition and did not participate in the intervention. The tests included paper and pencil and computer tasks. The cognitive constructs evaluated and the tasks associated with the constructions were: speed of processing, mental control, episodic memory, and visuospatial processing (Olchik et al., 2012). Stijntjes et al. (2013) performed a cross-sectional analysis within the longitudinal cohort of the Leiden longevity study in a sample of 500 individuals. They controlled memory function, attention, and processing speed. They also analyzed data with regression adjusted for age, sex, years of education, and additionally, for diabetes mellitus, cardiovascular diseases, alcohol consumption, smoking, inflammatory markers, and apolipoprotein E genotype. Robust standard errors were used to account for family relationships between children. Miller et al. (2013) performed neuropsychological tests, where three cognitive domains were compared (immediate memory, delayed memory, and language). Nishita et al. (2017) conducted a Longitudinal Study of Aging. The cognitive abilities of the participants were measured at the beginning using the short form of the Wechsler Adult Intelligence Scale of Japan, which includes the following tests: information (general knowledge), similarities (logical abstract thinking), completeness of images (perception visual and long-term visual memory), and the digit symbol (information processing speed). Zhu et al. (2017) used as a means of collecting information a survey evaluated by means of a self-reported scale. They used Cox proportional hazards models to examine the association of leisure activities with cognitive incidents; deterioration by controlling age, gender, education, occupation, residence, physical exercise, smoking, drinking, cardiovascular diseases and risk factors, negative well-being and physical functioning, and the initial MMSE score. Ritchie et al. (2013) used as a tool the control of the comparison of the IQ score in childhood and in adulthood at 70 and 79 years. According to Chang et al. (2012), the analysis was based on the NAHSIT data set 1999–2000. The Cox proportional risk models were used to evaluate the frequency of purchase in case of death between 1999 and 2008 with a possible adjustment of covariates. Gao et al. (2018) conducted a Longevity Study of Longitudinal Health of China. They adopted analyzes of life tables and discrete time risk models to examine the relationship between social participation and functional disability. For this, they defined social participation as the frequencies of participating in free time group activities (playing cards) and organized social activities, getting involved in informal social interactions (the number of brothers visited frequently), and participating in paid work. The extensive social participation was measured by a composite index adding the four types of social activities in which an older person was involved. The results determined that frequent card play was a protective factor for functional decline, where the relationship was partially mediated between cognitive abilities and positive emotions. Frequent participation in organized social activities is significantly related to a reduced risk of functional impairment, and this association was mediated between physical exercises and cognitive ability. The frequent visits of the brothers had a strong inverse relationship with the functional deterioration. However, it was observed that there was no significant association between paid work and functional decline.

From the analysis of the results obtained, several affirmations were seen, such as commitment, cognitive performance, and a greater sense of purpose which can serve as endophenotypes of longevity and a greater probability of survival. Similarly, several studies determined that learning new skills, the orientation to the search for novelty, learning new things, and keeping the mind engaged are important key to achieve a successful cognitive aging. Thus, training in the ability to solve problems of daily life improves cognition and is associated with greater performance of current memory. In addition, training generates changes in cognitive performance. In short, living a busy lifestyle is associated with better cognition.

Furthermore, it was concluded that there are other non-genetic aspects, such as learning new tasks, commitment, sense of purpose, occupation of the mind, cognitive performance, activities related to thinking, finding solutions to problems, etc., that favor the longevity of people in a successful aging.

## The Specific Wealth of Human Beings: Cultural Factors

Human beings are what we call a different being from the rest of living beings—we are a bio-psycho-social wholly. This is where the biological influences the psychological and the social, and where the psychological influences the biological. With this, we want to affirm that each person's personality (his psychology) and culture (his social self) modify the biology. In this way we can discern the influence of the biological, the psychological, and the social in each being and their differences with the rest of the living beings.

### The Evolution of “Our Genes”

The evolution of “Our Genes” if we now turn to the large numbers of the Human Genome Project, recently researched, these indicate that humans have about 25,000 or 30,000 Genes. By that, each one of us possesses about 100,000 million neurons and about 100 billion neuronal connections.

They also indicate that in the world, there are just over 6,500 million inhabitants and only 94 of 1,278 families of proteins in our genome are specific to vertebrates. Approximately 36% of the Worm Genome (7,000 genes) is the same as that of humans. The genetic difference between the Chimpanzee and the Human Being is only 1.3% our genes. Also, there is no genome difference between ethnic groups. The difference between the genomes of two people is around 0.2%, and it is responsible for each individual being unique and living. The difference between a man and a woman is only the difference of an “x” chromosome or “y.” That single and tiny different chromosome (“XX” or “XY”) makes us something (both) different and something (both) equal to men and women.

We start from the budget that almost only 20% of what we are is biology, and that the remaining 80% is the result of psychology and fundamentally of culture. It is the fruit of our brain. We modify the brain with the stimuli that come to us from outside. Let's quote that 80% is psychological, therefore, Life or disease depends on 20% of genes and 80% of life habits. Is it possible to intervene in our genes in favor of greater longevity (Borrell et al., 2011)?

María Blasco, director of the National Cancer Research Center of Spain has focused her research activity on telomeres, a structure of our chromosomes from which she and her team have obtained valuable information to further understand the process of life and disease and fundamentally of longevity.

In addition, environmental and psychological factors can affect the function of genes through what is called epigenetics (which acts as a switch to turn off or on, stimulate or slow down genes). That is, up to 20% of genes may be influenced by the psychological.

The length of telomeres depends on both genes—alterations in the telomerase enzyme gene can lead to telomeres that are much shorter than normal—and environmental factors. Smoking, obesity, anger, and negative position on life act negatively on telomeres. On the other hand, exercise, proper nutrition, and the psychology of optimism positively influence them.

A positive attitude can prevent depression, stress, insomnia, high cholesterol levels, and much more.

Being optimistic is directly related to enjoying good health. A positive attitude can prevent the development of diseases such as depression, stress, insomnia, inadequate cholesterol levels, etc. This is the conclusion that emerges from the study “Happiness and health perception” (2018), conducted by a group of researchers from the Complutense University of Madrid. On the other hand, it is also concluded that less happy people “tend to have more physical and psychological problems that affect them.”

In this sense, for example, the possibility of having depression in the group of less positive people is nine times greater than among those who are more positive. The probability of sleeping well is four times higher than among the most negative people.

According to this report, there is a two-way relationship between being happy and being healthy, as Carlos Chaguaceda points out, “people who feel happier and positive have a better perception of their health.”

And this association increasingly has more scientific evidence. This is confirmed by numerous studies: “Being more optimistic affects the nervous, neuroendocrine and immune system. For this reason, those who are happier, in general, suffer less cardio and cerebrovascular disorders and, as their immune system is reinforced, the chances of contracting diseases decrease” (Josep María Serra-Grabulosa, Department of Psychiatry and Psychobiology, University of Barcelona).

### The Search for Happiness

The problem arises in how to seek happiness. Although the brain has a natural propensity to have positive emotions, the authors of the report say, “It is necessary to stimulate it.” “*Happiness is worked; you cannot wait sitting at home.”* For example, those who perform sports on a regular basis are happy when they do it, and their brain generates a feeling that, although they get tired of exercising, they are happy. Likewise, when a painter projects himself in his work and gets excited in his construction of the painting he paints; his brain secretes serotonin and oxytocin and generates a sensation that makes him happy.

In short, we have to look for what makes us happy, and what makes us happy is to present ourselves creatively as positive human beings.

Furthermore, we should not forget the important role of social support or success and social triumph. The triumph, the achievement, the success, the creative and productive realization is fundamental for the people to feel with better state of health and help them to preserve, to a great extent, their level of satisfaction when they suffer a problem. Social support promotes happiness.

Obviously, there is not a single element that, in obtaining it, gives us happiness. It is necessary that science deepens in the way of favoring health from welfare. “Probably, there is the gene for happiness, but it is not just one, but there are several candidates that can provide this mental state and it is important to know how they are activated so that this happens.” At the moment, the gene that has more possibilities to be linked to happiness is **“the one that is related to serotonin”** substance that is activated mainly when we have positive experiences, when we feel creative and practice creativity both externally and internally.

### Positive Psychology and Positive Experiences

The main research of Positive Psychology has focused on discovering whether positive feelings and positive experiences leave their mark; as well as the way they leave their mark on our biological history in our physiology.

The relationship between positive emotions and health is very old; thus, for example, Galen (130–200), who is considered the father of modern medicine, in his treatise on tumors (De tumoribus) noted that “melancholic” women were more likely than “blood” to have cancer of breast.

That is, Galen highlights the importance of positive and/or negative health effects. Likewise, another historical, its equivalent in Spain, Maimonides (1135–1204) says in his “Guide to good health” that the doctor must make the greatest efforts so that all patients and individuals are always happy and that this produces health. He highlighted the preventive value of positive feelings and their therapeutic use.

Already in the nineteenth century, Darwin (1872) pointed out that emotions, both in people and animals, provide a signaling system necessary for survival and happiness.

### Relationship Between Emotions, Feelings and Positive Experiences, Health and Longevity

There are several researches and studies that confirm the importance of positive effects on longevity, but we have not found any that directly affect the level of studies and longevity.

#### The Oscars

Some studies related to positive experiences and/or prizes are referenced and can be a good foundation for what we are affirming. It's about the **Oscar awards** research. Redelmeier and Singh (2001) studied 235 deceased actors who had won an Oscar, and compared them, in longevity, with 527 actors who had participated in the same films, having been nominated without winning an Oscar; and finally, with 887 supporting actors also participating in the same films that had neither won an Oscar nor been nominated, and used as a control group. From this comparison, it appeared that the winners of an Oscar had lived 3.6 years longer than the nominees and 3.9 years more than the controls. In turn, Marmot (2004) reanalyzing these results found that the winners of several Oscars lived 2.7 years longer than those who only won one and 6.0 years more than the actors who had been used as a control group. Thus, having a great moment of positive affect that tilt the emotional balance in favor of the latter seems to influence not only health but to achieve greater longevity.

#### The Study on the Nuns

Another very significant study is the Positive Emotions in Early Life and Longevity: Findings from the Nun Study; Deborah D. Danner, David A. Snowdon, and Wallace V. Friesen, University of Kentucky.

It is a study made with handwritten autobiographies from 180 Catholic nuns, composed when the participants had an average age of 22 years. They were asked and scored on emotional content and analyzed the answers in relation to the survival of the same at ages 75–95.

After analyzing the data, a strong association was found between the positive emotional content in these writings and the risk of mortality in the elderly (*p* < 0.001).

The quartile ranking of positive emotion in early life increased, presenting a gradual decrease in the risk of mortality that turned out to be a difference of 2.5 times between the lowest and highest quartiles. As a conclusion, it was empirically deduced that the highest positive emotional content in autobiographies, in early life, is strongly associated with longevity in 6 decades later.

#### The Positive Perception

Another longitudinal study that lasted more than 20 years, conducted by Levy et al. (2002) showed that older people with more positive perceptions of their aging in basic feeling (when they were 50 or older) lived longer (an average of 7.6 years more) than those who presented more negative perceptions about the process of becoming an elder. Therefore, the promotion of well-being in this vital segment is especially necessary (Chiang, 1984).

#### An Empirical Result

The results of an investigation carried out by S. MacManus in 1979 on the duration of the life of the Italian artists during the Renaissance indicate that the average age of death of these artists was more long-lived than that of their contemporaries.

The longevity of 218 artists was studied (born between 1,250 and 1,550). Their average age of life was 63.03 years when the average age was 53–56 years.

Its curve of average duration of the life of these artists was identical to that of the English men of 1,881 (five centuries later).

We see then that positive life experiences contribute to health, health to happiness, and happiness, directly or indirectly, to feel better and feeling a better one is better to achieve longevity.

Undoubtedly, after the analysis of the various investigations presented, we can indicate that the positive feelings are like Endogenous Opiates (Serotonin and Oxytocin) that produce a great benefit to health and in longevity. The direct effects of positive affect on immune function are investigated in this regard. From several investigations, it is hypothesized that a positive affective style would be associated with a greater activation of the immune system, while a negative style would be associated with a less or no activation of the immune system.

As a conclusion of this line of work, it must be said that we do not have conclusive evidence that positive affect provides a sustained increase in immune function. What the research does support is that the chronic absence of positive affect is related to immune deficiency.

Positive emotions shorten the duration of cardiovascular activation produced by negative emotions or, at least, at the level of symptoms, influence less pain, better health, and provide greater security to the disease by providing more internal security and more social support. Positive emotions have a positive effect. They stimulate and develop the positive self-concept that enables the person to feel valuable and capable; use humor as a positive reaction; feel good about yourself and others; appreciate the effort of living and committing; perceive changes and difficulties as challenges to be solved and understood; always think of the good wishes, sincerity, and truthfulness of others and be open to cultural interests, to change, ideas and values different from your own; and above all make the effort to achieve goals of realization.

In addition to the direct effects, experiences, and positive obligations also indirectly influence health through: the enhancement of activity behaviors, the need to live, and these in turn influence health. Likewise, the need to live facilitate cognitive and social processes that also result in better health (Maldonado-Briegas et al., 2019).

In short, emotions and positive positioning facilitate the creation of social relationships and friendship, which in turn allow the person to acquire social resources that can be used later, when needed.

If all that is so, can we find something empirical and real to prove it? Our answer and I think that almost all of you is that YES.

## Cognitive Development and Longevity

It seems that a variable that affects determinantly in longevity is cognitive development. Studies of the last century, such as that of Alciati (1987) (Table 1) advanced that “*as the human being has developed his brain, his cognitive capacity has been progressing in longevity.”*

If we analyze the binomial cognitive development and longevity, we will observe that there are several studies that relate these two concepts. Some of them even relate it to the level of academic studies reached by people, as well as their performance as teachers. Lucchese et al. (2018) affirm that people, both men and women, who have more levels of education and practice daily as teachers live for an average of more years. The study, focused on the groups aged 60 and over collected in the INE (National Institute of Statistics) Census of 2018, concludes that having a higher level of education means being more positive and happy and living longer. Likewise, people performing a teaching task and developing this activity with satisfaction leads them to maintain an open and satisfactory cognitive life; therefore, the conjunction of a higher level of studies with a life lived with happiness helps to live more years.

It is understandable, therefore, that the performance of higher level studies at advanced ages favor longevity. In fact, learning new things and keeping the mind compromised can be an important key to successful cognitive aging (Park et al., 2014). Undoubtedly, the effort and commitment required by the academic formation establishes a sense of purpose and a clear objective in the life of the student that favors longevity, and (Windsor et al., 2015) greater sense of the purpose was associated with a greater probability of survival and longevity. Thus, participating in learning new skills improves episodic memory in older adults, improves cognition in relation to participation in social or challenging activities, and helps to live longer (Chan et al., 2016).

Cognitive development, the process by which the human being is acquiring knowledge through learning and experience, is established in different facets of life and in different ways, such as through the ability to solve daily problems. Although these problems differ with age along with the underlying processes, they increase longevity (Chen et al., 2017).

It is therefore understandable that man, as a social, dynamic, active, creative, and complex entity, is linked to a social participation. Thus, the conservation or improvement of cognitive development through social activity, regular participation in group leisure activities, organized social activities, and informal social interactions in particular can have beneficial effects on the functional health of adults (elderly people) through behavioral and psychosocial pathways (Gao et al., 2018).

## Studies on Longevity

Previously, the results of previous investigations have been commented (Lucchese et al., 2018). Thus, the different demographic studies show that there is a greater longevity of the population in relation to the whole population, and that among the many variables that influence significantly, one of them is the development in the educational levels and the corresponding cognitive development.

According to the study carried out on cognitive development and longevity (Lucchese et al., 2018), the level of studies is a significant variable that promotes longevity. The sample used was the totality of the Spanish population according to the INE (National Statistics Institute) Census of 2017.

The main conclusions reached were that people, both men and women, who had more levels of education lived on average more years. The data indicated that the levels of studies were a significant variable with respect to the years of life. People with lower educational levels died earlier. Therefore, life expectancy is directly related to educational levels.

Returning to the study carried out, the demographic indicators, specifically data from the INE Continuous Register (on January 1, 2017), indicated that in Spain, there were 7,754,956 older people (65 and over), 16.67% of the total the population (46,528,024). The proportion of octogenarians represented 5.09% of the population, and according to some studies, this group will continue to grow thanks to technological and medical advances and the implementation of active aging programs (Abellán and Pujol, 2016).

**Figure 8:**
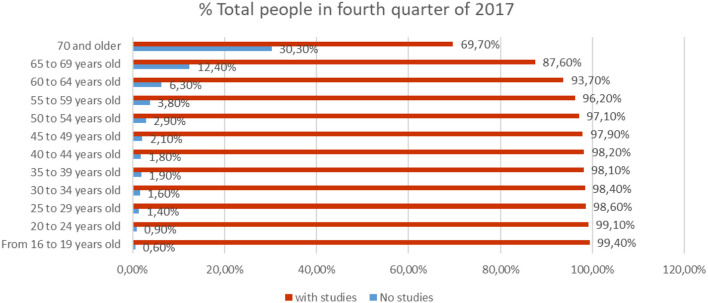
% Total people who have studies according to age (source: prepared by the authors based on INE data).

**Figure 9:**
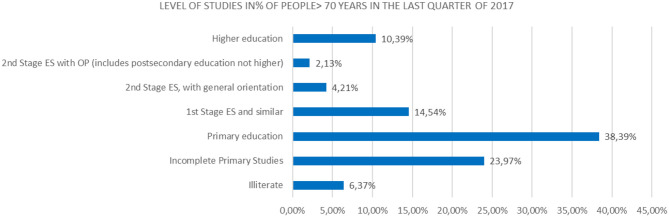
Level of studies in % of people over 70 years of age in the last quarter of 2017 (source: prepared by the authors based on INE data).

According to the comparative analysis between people who have no studies (illiterate and people with incomplete primary education), and people who, if they have studies, referenced to the last 4 months of 2017, according to data from the INE. The total % of people who have studies is above 69% in people over 70 years, with 87.6% among those between 65 and 69 years old, and over 90% in people under the age of 65 years, while they only live a percentage of 30% of those who do not have studies (Figures 8, 9).

**Figure 10:**
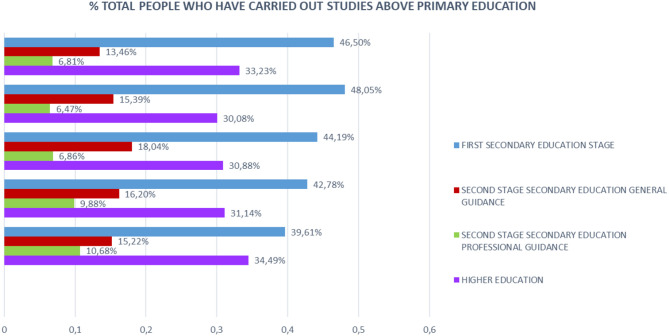
Level of studies in % of people over 70 years of age in the last quarter of 2017.

If we make a comparison in the level of studies over 50 years, focusing on the partial % of people who have done any study over primary education, we will note that the % of people with higher education is higher in people >70 years, which, in people above 55 years of age, indicates the life and mortality rates after 65 years of age in relation to the studies (Figure 10).

In the same analysis of studies based on sex, we observe that 74% of men of 70 or more years have studies, compared to 26%, who did not finish them.

**Figure 11:**
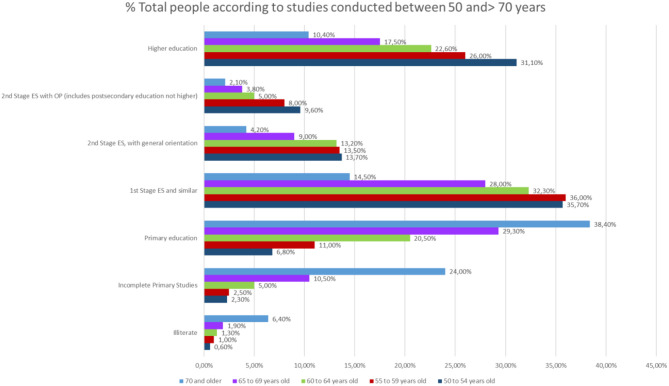
The % of people between the ages of 50 and 70, according to their level of education (source: own elaboration based on INE data).

On the other hand, in women, aged 70 or older, 66.4% have studies compared to 33.6% which did not finish them. The following graph shows a comparison between the number of people in % who have done any study of any level and the number of people who have not done any study or have not completed it, in ages over 50 years (Source: INE) (Figure 11).

The distinction between the different levels of studies for those who finished their studies is significant, since those people over 70 with higher education levels surpass those in the Second Stage of secondary education with job or general orientation.

In fact, analyzing that segment of people with ages over 50 years, it is observed that there are more people with studies of Second Stage of Secondary or Higher Education, 16.7%, compared to those who only have studies of the First Stage of Secondary, 14.5%.

**Figure 12:**
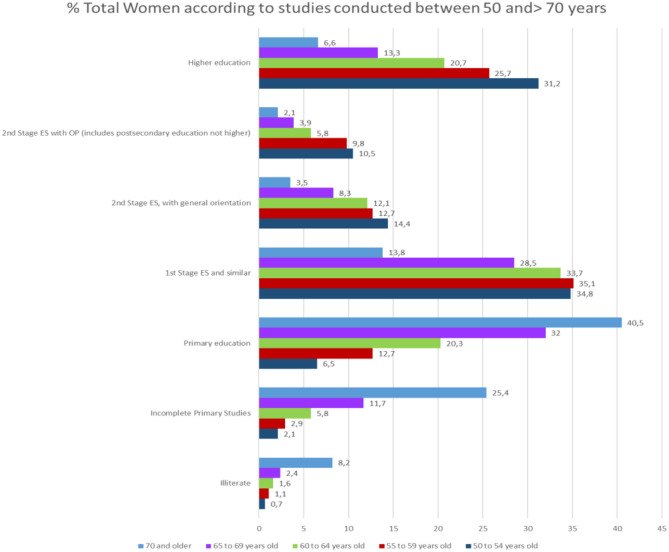
The % of women between the ages of 50 and 70, according to their level of education.

The difference in the level of studies in men and women becomes relevant in people older than 65 years of age. In people over 65 and younger than 69% of people with higher education is 22.4% in men and 13.3% in women, while in people over 70 years, 15.5% of men have higher education compared to 6.6% of women (Figure 12).

According to the data collected by the INE, the Mortality based on the level of Studies for the year 2016 was reflected in Table 2 (Collected in Table 2 of this document).

**Table 2:** Mortality in people according to the level of studies in 2016 according to the data collected by the INE.

		**Illiterate**	**Incomplete primary studies**	**Primary education**	**First stage of secondary and similar education**	**Second stage of secondary education with general guidance**	**Second stage of vocational secondary education**	**Postsecondary education not higher**	**Vocational, plastic and design arts and sports education of higher and equivalent degree**	**University degrees of 240 ECTS credits, diplomas, own university degrees of expert**	**University degrees with more than 240 ECTS credits, bachelor's degree and similar**	**Masters, specialties in Health Sciences by the residence system and similar**	**University Doctorate**	**Not recorded**
Both genders	407,158	12,948	98,097	139,312	79,681	22,350	9,852	22	7,938	13,790	12,499	812	2,005	7,852
	Percentage	3.18%	24.09%	34.22%	19.57%	5.49%	2.42%	0.01%	1.95%	3.39%	3.07%	0.20%	0.49%	1.93%
Men	206,801	3,104	42,553	66,987	44,909	13,796	6,049	12	5,897	8,004	8,747	529	1,596	4,645
	Percentage	2%	21%	32%	22%	7%	3%	0%	3%	4%	4%	0%	1%	2%
Women	200,357	9,844	5,544	72,325	34,772	8,581	3,803	10	2,041	5,796	3,752	283	409	3,207
	Percentage	5%	28%	36%	17%	4%	2%	0%	1%	3%	2%	0%	0%	2%

It was note as indicated in the census Las Fuentes de información to assign educational level to the entire population (*National Institute of Statistics-INE). There were Displacement statistics Assignment of educational level to death records of 2015 and obtaining method and warnings to users (December 2016)*.

### To Obtain the Level of Studies Reached by a Person, the Following Sources Have Been Used

∙ Register: *The school or academic title is a variable of registration that must be collected by municipal councils in its municipal register and, therefore, is included in the INE's census database, although the INE does not disseminate, through the Statistics of the Continuous Register; the distribution of the population by this variable. The incorporation in the Register is related to the formation of the Electoral Census that is carried out from the census information. Thus, in the continuous management of the Register, this information is collected from the town councils and is purified with the information received semiannually by the Ministry of Education, Culture and Sports on titles issued*.

It is important to note that the Municipal Register management standards allow two types of classifications by municipalities of this variable. However, there is no information regarding the school title with the same level of detail for all people. Thus, 33.8% of the population is encoded with the simplified classification, which is the one that appears in the electoral census (4 aggregate levels); while 66.2% has some value of the complete classification (13 detailed levels) (Table 2, collected in Table 2 of this document).

**Figure 13:**
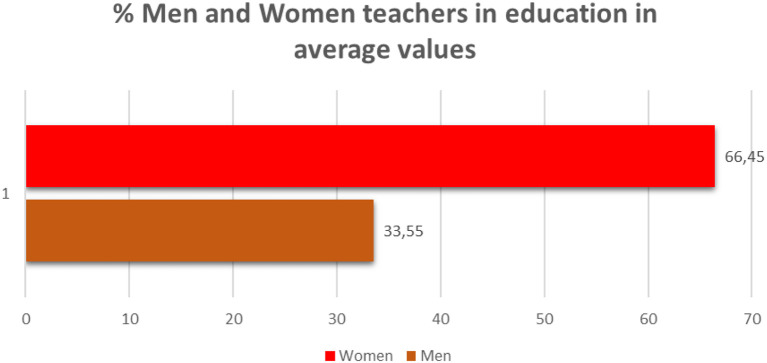
Percentage of men and women teachers in the academic year 2016–2017 in average values (source: own elaboration based on data from the Institute for Women).

**Figure 14:**
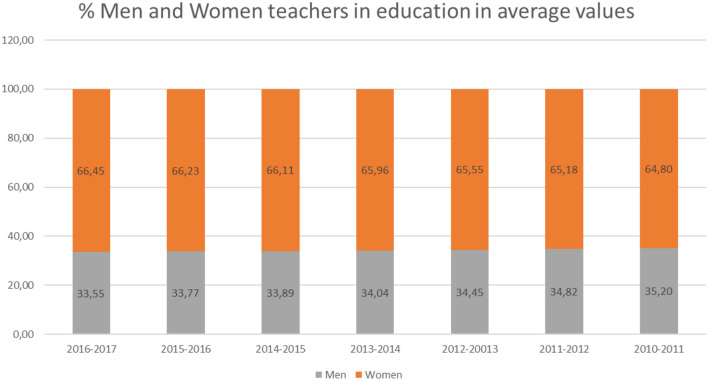
Percentage of men and women teachers in different academic courses in medium terms (source: own elaboration based on data from the Institute for Women).

If we take into account data from the Women's Institute of the Ministry of Health, Social Services and Equality, in the academic year 2016–2017, on average, 66.45% of teachers in Spain were women (Figure 13). This percentage has been gradually increasing every year (Figure 14).

**Figure 15:**
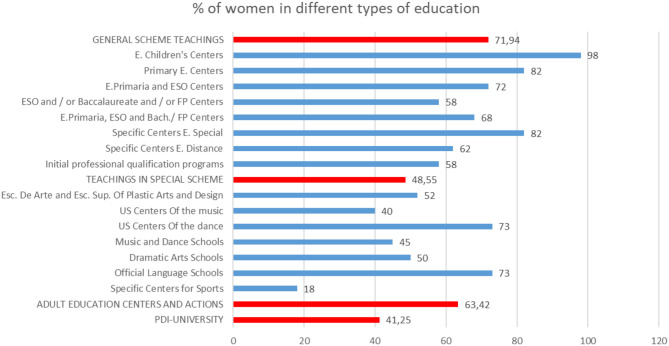
Percentage of women teachers in different specialties during the academic year 2016–2017 (source: own elaboration based on data from the Institute for Women).

Among the specialties and levels of education in which the presence of women in the classrooms in the academic year 2016–2017 was leading, they were in the general education courses and in adult education centers and activities (Figure 15).

**Figure 16:**
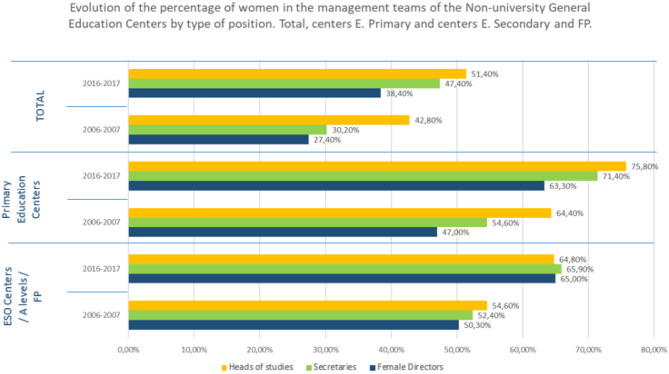
Source: Statistics of Non-University Teachings. Teacher series, MEFP.

However, the evolution in managerial positions of women in education has been increasing over the last decade as reflected in the following graph (Figure 16).

**Figure 17:**
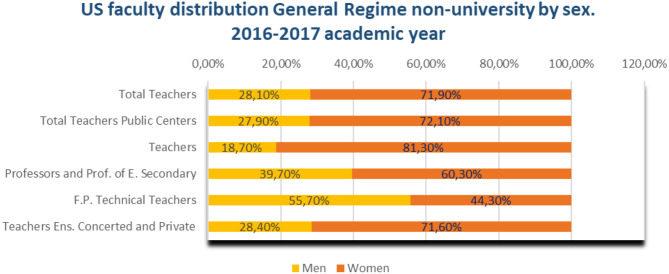
Distribution of teachers in the USA General non-university regime by age and sex during the 2016–2017 academic year (source: data and figures report, School year 2018/2019, Ministry of Education and Vocational Training).

In the 2016–2017 academic year, the distribution of teaching staff in general non-university education was led by women (Figure 17). 5.5% of the active population is dedicated to non-university education from the age of 60.

**Figure 18:**
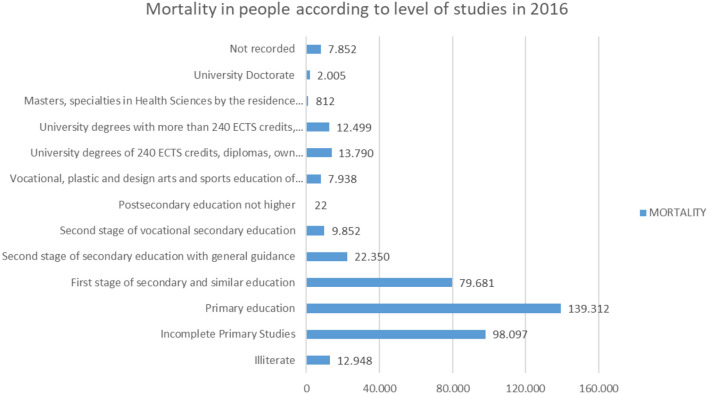
Mortality of people in 2016 according to their level of education.

**Figure 19:**
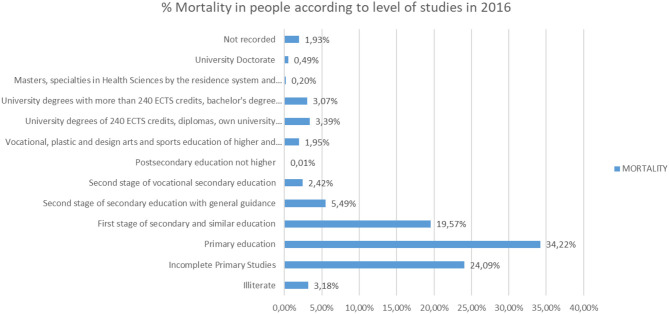
Mortality of people in 2016 in % according to their level of education.

On the other hand, if we analyze the relationship between the level of studies and mortality, according to the data collected in INE (2016) (the tabulated values are included in Table 2), we will observe the following (Figures 18, 19).

It shows that, of 407,158 deaths in 2016, the highest number of deaths (139,312), 34.22%, were those whose highest levels of study were in Primary Education, followed by those who did not have primary education complete (98,097), that is, 24.09%.

**Figure 20:**
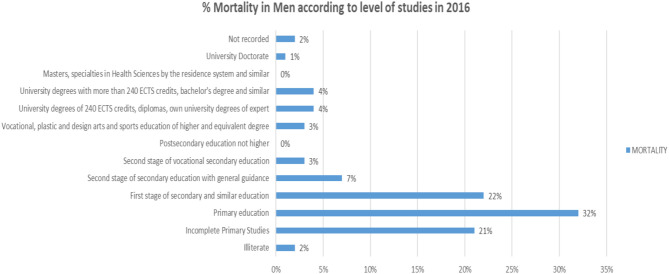
Mortality of men in 2016 in % according to their level of education.

On the other hand, if we analyze the % of total deceased men according to the level of studies (Figure 20), we will observe that the highest number of people who died in 2016 were those who had Primary Education, incomplete primary education, and First stage of secondary education or similarly.

**Figure 21:**
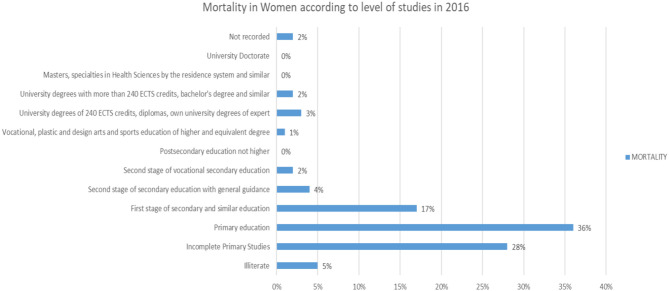
Mortality of women in 2016 in % according to their level of education.

The same happens with the female sex (Figure 21).

**Figure 22:**
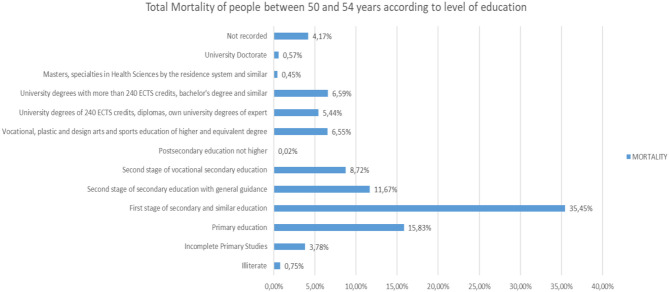
Number of people deceased between 50 and 54 years according to their level of education.

If we analyze the different Mortality Rates according to the level of studies in people between 50 and 54 years old (Figure 22), we will observe that 35.45% have the First Stage of Secondary Education and similar, and Primary Education as the highest level of studies, 15.83%.

**Figure 23:**
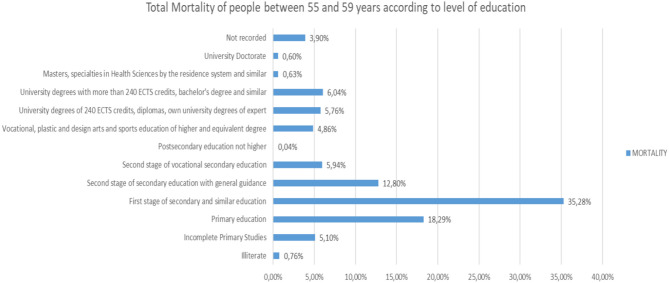
Number of deceased people between 55 and 59 years of age according to their level of education (source: own elaboration based on INE data).

If we analyze the different Mortality Rates according to the level of studies in people between 55 and 59 years old (Figure 23), we will observe that 35.28% have Higher Secondary Education Stage and similar, and Primary Education, as the highest level of studies, 18.29%.

**Figure 24:**
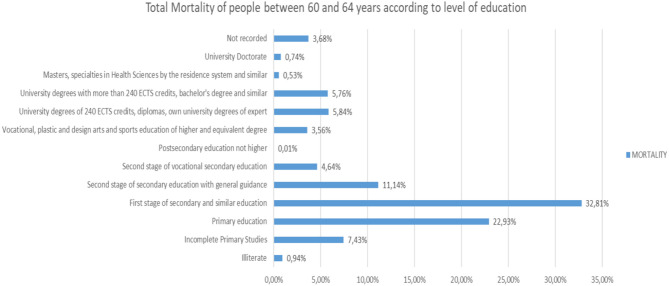
Number of deceased people between 60 and 64 years of age according to their level of education (source: own elaboration based on INE data).

If we analyze the different Mortality Rates according to the level of studies in people between 60 and 64 years old (Figure 24), we will observe that 32.81% have the Higher Education Stage and the like, and Primary Education, as the highest level of studies, 22.93%.

**Figure 25:**
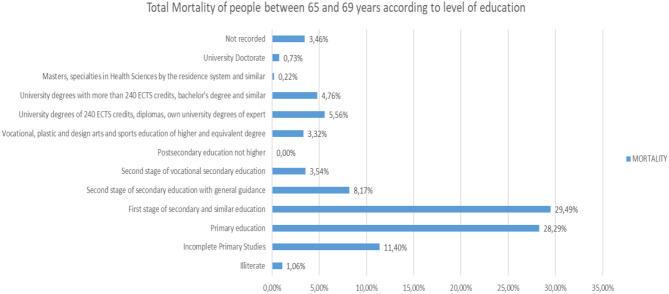
Number of people deceased between 65 and 69 years of age according to their level of education (source: own elaboration based on INE data).

**Figure 26:**
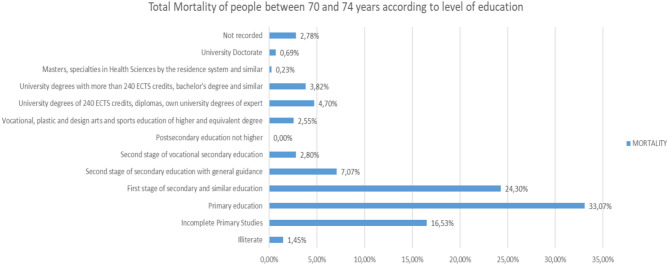
Number of people deceased between 70 and 74 years according to their level of education.

**Figure 27:**
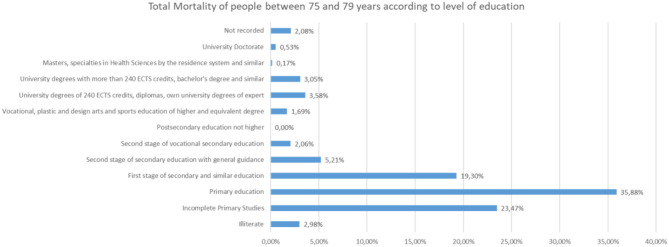
Number of people deceased between 75 and 79 years according to their level of education.

**Figure 28:**
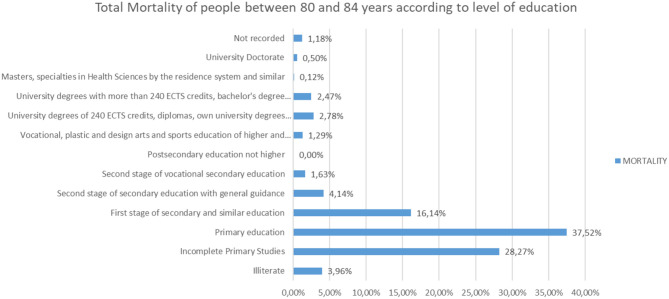
Number of people deceased between 80 and 84 years according to their level of education.

**Figure 29:**
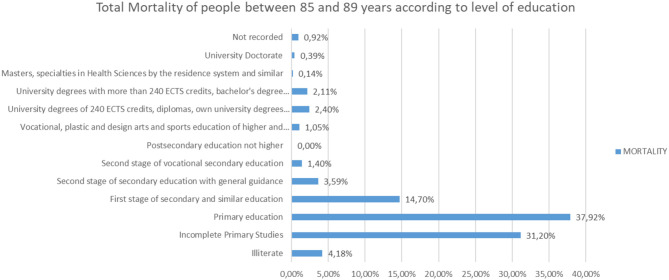
Number of people deceased between 85 and 89 years according to their level of education.

**Figure 30:**
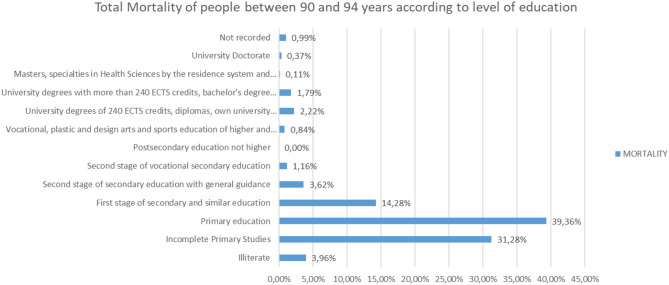
Number of people who died between 90 and 94 years of age according to their level of education (source: own elaboration based on INE data).

**Figure 31:**
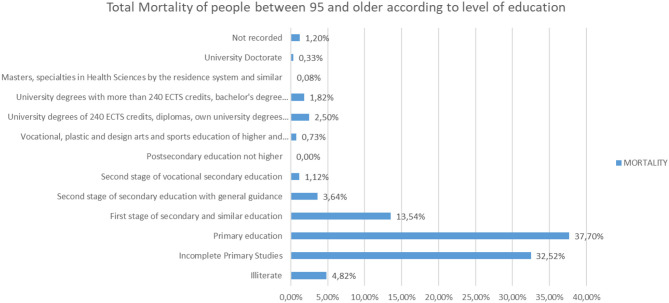
Number of people deceased between 95 and more years according to their level of education (source: own elaboration based on INE data).

Analyzing the rest of the classes by ages from 65 to more than 95 years, the same trend is observed (Figures 25–31).

## Conclusion

The increase in people's life expectancy (longevity) is linked to several variables; some of these variables are determinant than others. Focusing on the literature review and the studies analyzed in this document, we conclude that certain psychological variables linked to psycho-socio-cultural aspects favor the increase in longevity.

Attitudes such as commitment, maintaining an active life, related to cognitive performance and a greater sense of purpose, can serve as predictors of longevity. This means that learning new skills, keeping the mind occupied, motivated by the search for novelty, and by solving problems of daily life are important key to maintaining a successful cognitive aging. Likewise, the realization of activities with positive mental states generates health benefits. In fact, positive emotions and experiences help prevent diseases, and are often predictors of health and longevity.

Similarly, people with higher levels of study manage to live longer. It was affirmed by Ritchie et al. (2013), which conclude in their study, that the increase in education significantly improves cognitive abilities of later life. Affirmation also ratified by Lucchese et al. (2018) which assure that the levels of studies are a significant variable with respect to life expectancy is directly related to the levels of studies.

Therefore, academic training, if we understand it as a training of memory, could be interpreted as a feasible non-pharmacological intervention that could bring a positive change in performance in older adults who face cognitive impairment (Olchik et al., 2012).

## Author Contributions

All authors contributed at the same level for the research project, data collection and analysis, and paper writing.

## Conflict of Interest

The authors declare that the research was conducted in the absence of any commercial or financial relationships that could be construed as a potential conflict of interest.

## Footnotes

^1^INE: Instituto Nacional de Estadística. (Statistics National Institute) (https://www.ine.es/).

## NOMENCLATURE INDEX

### UAM (Autonomous University of Madrid)

The UAM_Biblioteca works to support the development of research, teaching and study, providing access to necessary information resources, specific to this University or outside it. https://biblioguias.uam.es/psicologia/psicologia/bdatos

### Pepsic

The portal of Electronic Newspapers of Psychology (PePSIC) is a source of the Virtual Health Library. Psychology of the Latin American Union of Psychology Entities (BVS-Psi ULAPSI) and result of the association between the National Entities Forum of Brazilian Psychology (FENPB), Dante Moreira Leche Library of the Institute of Psychology of the University of São Paulo (IP/USP) and the Latin American and Caribbean Center of Information in Health Sciences—BIREME, which provided the methodology—Scientific Electronic Library Online (SciELO)—model of electronic publication of newspapers to developing countries.

http://pepsic.bvsalud.org/scielo.php?lng=es

### Scielo

Scielo España is a virtual library formed by a collection of Spanish scientific journals of health sciences selected according to pre-established quality criteria. The SciELO project is the result of cooperation between BIREME (Latin American and Caribbean Information Center in Health Sciences) and FAPESP (Fundação de Amparo à Pesquisa do Estado de São Paulo). In Spain, it is being developed by the National Library of Health Sciences, thanks to the collaboration agreement established in PAHO/WHO and the Carlos III Health Institute.

http://www.scielo.org/php/index.php?lang=es

### Lilacs

Latin American and Caribbean Literature in Health Sciences. LILACS acronym, it is a database of online bibliographic information for health sciences. In general, it covers the entire literature of the area, produced by authors from Latin America and published in the countries of the region since 1982. LILACS is maintained by the Latin American and Caribbean Center for Health Sciences Information (also known as BIREME), located in São Paulo, Brazil. As in MEDLINE, which was developed by the National Library of Medicine of the USA. UU., Includes bibliographic references of documents that have been published in a set of scientific and medical publications of the region, and that are not covered by MEDLINE.

http://lilacs.bvsalud.org/es/

### Psycinfo

Bibliographic database of the American Psychological Association that contains citations and summaries of journal articles, books, doctoral theses, and reports.

https://www.apa.org/pubs/databases/psycinfo/coverage.aspx

### Pubmed

PubMed is a search engine for free access to the MEDLINE database of citations and abstracts of biomedical research articles. Offered by the National Library of Medicine of the United States as part of Entrez. MEDLINE has around 4.800 journals published in the United States and in more than 70 countries around the world from 1966 to the present.

https://www.ncbi.nlm.nih.gov/pubmed/

### Teseo

Teseo is the database on the internet that allows you to learn about doctoral theses defended in Spanish universities since 1976.

https://www.bvsspa.es/profesionales/bbdd-y-otros-recursos/recursos/teseo-base-de-datos-de-tesis-doctorales

### Dialnet

It is a library cooperation project that started at the University of La Rioja. It is constituted as a portal that collects and provides access primarily to documents published in Spain in any language, published in Spanish in any country or dealing with Hispanic issues. The contents include journal articles, chapters of collective monographs, doctoral theses, books, etc. Its wide coverage makes Dialnet the largest database of Hispanic scientific articles accessible for free on the Internet.

https://dialnet.unirioja.es/
